# Next Generation Sequencing to Determine the Cystic Fibrosis Mutation Spectrum in Palestinian Population

**DOI:** 10.1155/2015/458653

**Published:** 2015-01-26

**Authors:** O. Essawi, M. Farraj, K. De Leeneer, W. Steyaert, K. De Pauw, A. De Paepe, K. Claes, T. Essawi, P. J. Coucke

**Affiliations:** ^1^Department Master Program in Clinical Laboratory Science, Birzeit University, 9700 Birzeit, State of Palestine; ^2^Center for Medical Genetics, Ghent University, 9000 Ghent, Belgium

## Abstract

An extensive molecular analysis of the CF transmembrane regulator (*CFTR*) gene was performed to establish the *CFTR* mutation spectrum and frequencies in the Palestinian population, which can be considered as an understudied population. We used a targeted Next Generation Sequencing approach to sequence the entire coding region and the adjacent sequences of the *CFTR* gene combined with MLPA analysis of 60 unrelated CF patients. Eighteen different CF-causing mutations, including one previously undescribed mutation p.(Gly1265Arg), were identified. The overall detection rate is up to 67%, and when we consider only CF patients with sweat chloride concentrations >70 mEq/L, we even have a pickup rate of 92%. Whereas p.(Phe508del) is the most frequent allele (35% of the positive cases), 3 other mutations c.2988+1Kbdel8.6Kb, c.1393-1G>A, and p.(Gly85Glu) showed frequencies higher than 5% and a total of 9 mutations account for 84% of the mutations. This limited spectrum of CF mutations is in agreement with the homozygous ethnic origin of the Palestinian population. The relative large portion of patients without a mutation is most likely due to clinical misdiagnosis. Our results will be important in the development of an adequate molecular diagnostic test for CF in Palestine.

## 1. Introduction

Cystic fibrosis (CF) is a severe life threatening genetic disease most common among Caucasians with an incidence ranging from 1 in 2500 to 1 in 3600 [1]. CF is inherited in an autosomal recessive way and the cystic fibrosis transmembrane conductance regulator gene (*CFTR*), located on chromosome 7q31.2 [2], has been identified as the responsible gene encoding a transmembrane protein that functions as a chloride channel and a regulator of other channels across the epithelial cell membrane. The defective protein impairs water movement across epithelia leading to formation of viscous mucus that obstructs the airways of the lungs and ducts of the pancreas. CF is characterized by progressive lung disease, pancreatic dysfunction, elevated sweat electrolytes, and male infertility [3].

So far, more than 1900 different* CFTR* mutations have been reported [4]. Although most mutations are rare, the three-base-pair deletion p.(Phe508del) is most common in the Caucasian population affecting about 70% of the patients whereas in the Jewish population the p.(Trp1282^*^) is the most prevalent with a frequency of 60% [5], clearly indicating that the occurrence of mutations is highly population specific. For many ethnic or geographic populations, the mutation spectrum has been determined [6–15].

Recently, CF has been diagnosed in the Middle East ranging from 1 in 2,500 to 1 in 16,000 with different mutation frequencies according to the ethnic origin of populations [16]. However, reliable information about the frequency of CF among the Palestinians is still lacking and the spectrum and nature of mutations have not been documented yet hampering molecular diagnostics. A good insight into the nature and frequency of the mutations in a specific population is a prerequisite to set up adequate and cost-effective molecular diagnostics.

The aim of this study was to determine the CF mutation spectrum among the Palestinian patient population. Samples from 60 unrelated CF patients residing in the West Bank and Gaza were collected and their respective CF mutations were determined. Consequently, the mutation spectrum was compared with other ethnic groups residing in the Arabic population.

## 2. Materials and Methods

### 2.1. Patients and Sample Collection

A total of 60 unrelated Palestinian CF patients, 19 of them residing in the West Bank and 41 residing in Gaza, participated in this study of which 34 are males and 26 are females. Most of the participants (97%) were children less than 18 years old. The criteria for inclusion in this study were based on the clinical diagnosis. Typical pulmonary and/or gastrointestinal tract manifestations and/or elevated sweat chloride values (>60 mEq/L, Table 1) were the main criteria. Whole blood (3 mL) was collected in EDTA vacutainer tubes (BD). Participation in this project was based on the free will of the participants. Signed consent was obtained from each participant and/or the guardian.

### 2.2. DNA Extraction and Polymerase Chain Reaction (PCR)

Genomic DNA was extracted and purified from whole EDTA-blood by the automated extraction apparatus Autopure LS (Qiagen) using the PureGene DNA Purification Kit (Qiagen).

Amplification of the coding region and flanking introns of the* CFTR* gene was conducted using the 2720 thermal cycler (Applied Biosystems). A total of 28 sets of primers were developed, flanking at least 25 intronic nucleotides away from each of the 27 exons of the* CFTR* gene. Primers are found in supplemental Table 1  (see supplemental Table 1 in Supplementary Material available online at http://dx.doi.org/10.1155/2015/458653). Amplification was performed with 2.5 *μ*L of the purified DNA (50 ng/*μ*L) template in a total of 10 *μ*L reaction mixture. The complete mix constituted the following components: 5 *μ*L of 2X KAPA2G Robust Hot Start Ready Mix (Kapa biosystems), 1.25 *μ*L upstream primer (0.1 *μ*M), and 1.25 *μ*L downstream primer (0.1 *μ*M). The following amplification conditions were used: 95°C for 3 min and then 95°C for 15 sec, 60°C for 10 sec, and 72°C for 15 sec, for 32 cycles, with final extension at 72°C for 1 min. The LabChip GX (PerkinElmer, USA) capillary electrophoresis was used to assess PCR product.

### 2.3. NGS Sequencing

For each patient, all PCR products were pooled equimolary before they entered the Nextera sample preparation protocol (Nextera XT DNA Sample Prep Kit (Illumina, Inc., San Diego, CA)), followed by 250 bp single-end sequencing on a MiSeq instrument (Illumina, Inc., San Diego, CA). All 60 samples were labeled using 60 different index tags (Nextera, Epicentre Biotechnologies).

### 2.4. Data Analysis

Reads were aligned to the human genome (hg19/GRC37) using the CLCBio software package (CLC Genomics Workbench 6.0.2). All exons with a coverage lower than 20 were analysed by Sanger sequencing. We first filtered all variants classified as deleterious according to the* CFTR* mutation database (ENST00000003084) [4]. To identify new mutations the data were filtered against dbSNP135 (MAF > 1%). For novel variants, causality was assessed using* in silico* prediction software (Alamut, Interactive Biosoftware, Rouen, France).

### 2.5. Mutation Confirmation

Mutations were confirmed with the INNO-LiPA* CFTR*17 and* CFTR*19 kit (Fujirebio, Europe) which provides probes for the 35 most frequent* CFTR*-related mutations worldwide or by Sanger sequencing. Direct sequencing of the PCR product was performed on an ABI3130XL sequencer (Applied Biosystems, Inc., Foster City, CA). For the larger deletions or insertions and for all patients with only one or no mutation, multiple ligation-dependent probe amplification (MLPA) analysis was applied (MRC-Holland, SALSA MLPA KIT P091-D1* CFTR*).

## 3. Results 

### 3.1. NGS Analysis

We applied an NGS screening strategy to efficiently identify causative mutations in the 60 unrelated Palestinian patients with a clinical diagnosis of CF. Approximately, 98% of all reads on the MiSeq were successfully mapped to the reference genome. The overall mean read depth in the target area was 344x. A read depth of 10x for 92% of the bases and 20x for 90% of the bases was obtained. Patient P9 had the best coverage with all amplicons covered with at least 20x whereas patient P7 had the lowest number of amplicons (13) with a coverage higher than 20x (data not shown).

### 3.2. Identification of Causative Mutations

**Figure 1:**
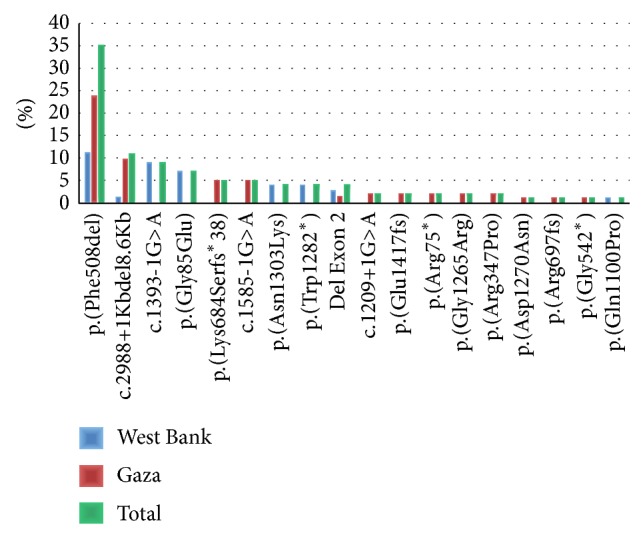
*CFTR* mutation spectrum among Palestinians in Gaza and West Bank.

We identified 17 different mutations in 40 patients which have previously been described as CF-causing mutations, including 3 splice sites, 5 missense mutations, 4 frame shift mutations, 3 stopcodons, and 2 exon spanning deletions (Table 2). A homozygous exon-spanning deletion was suspected when we were unable to amplify the exons 19, 20 and 21 by PCR and consequently we did not obtain any read for these amplicons by NGS. The presence of the deletion was confirmed by MLPA analysis. In addition we identified one novel potentially deleterious homozygous missense mutation p.(Gly1265Arg) (phyloP: 5.53, Grantham dist.: 125, Sift: Deleterious, Mutation Taster: disease causing). The NGS approach does not allow, at least with the PCR-enrichment approach applied in this strategy, detection of large heterozygous deletions or insertions. Therefore, we further investigated the remaining 19 negative patients and the 3 heterozygous patients for the presence of deletions/insertions using MLPA analysis. This revealed two heterozygous deletions in patients P24 and P49. In total, we were able to detect 81* CFTR* mutations on a total of 120 alleles from 60 CF patients. Mutations were compared between CF patients originating from the West Bank and those from Gaza, as both regions are physically divided over 66 years. Data are represented in Figure 1.

## 4. Discussion

This is the first study to investigate the* CFTR* mutation spectrum in the Palestinian population that was conducted on 60 unrelated CF patients residing in the West Bank and Gaza, Palestine. We identified in 41 of these probands 81* CFTR* mutations of which 18 are different. All patients are homozygous or compound heterozygous, except patient 18, in whom we identified only one single mutation. This may be explained by the fact that the technology used in this approach does not allow detection of deep intronic mutations. The five most common mutations are p.(Phe508del) followed by the deletion of exons 19–21 (c.2988+1Kbdel8.6Kb), c.1393-1G>A, p.(Gly85Glu), and p.(Lys684Serfs^*^38), representing 67% of all mutations (Figure 1). The homozygous p.(Gly1265Arg) mutation, which we identified in an 8-year-old male from Gaza, was never reported before. Comparing our results with a study conducted in Israel by Laufer-Cahana et al. [17] among Israeli Arab patients showed similar rates for mutations p.(Phe508del) (34%), p.(Gly85Glu) (8%), and p.(Lys684Serfs^*^38) (8%) and the deletion of exons 19–21 deletion (c.2988+1Kbdel8.6Kb) (13%), confirming the common origin of both populations. Interestingly, whereas the rate of the p.(Phe508del) mutation is similar to that in the neighboring Lebanese CF population and the Jewish CF populations from Balkani (Turkish and Greeks) and Ashkenazi origins [1, 15], the deletion of exons 19–21 (c.2988+1Kbdel8.6Kb) was only present in Palestinian Arabs which may indicate that this mutation is a founder mutation among this population [18, 19]. The two mutations p.(Gly85Glu) and c.1585-1G>A, which were identified in this study, were also found among the Jewish CF populations from Balkani (9.5%) and Ashkenazi (1%) origins, respectively [15]. On the other hand, one of the most frequent mutations in the Jewish CF population p.(Trp1282^*^) (31%) was found to be less frequent in this study (4%), similar to other Arabic CF populations such as Tunisians (4.4%) and Algerians (4.2%) [8, 9, 15]. The rate of the p.(Asn1303Lys) mutation was about the same in Ashkenazi Jews and the Palestinian CF population tested in this study (5%), as well as in the Iranian (4.3%) and Tunisian (6.6%) CF populations [8, 11, 15]. Designing an allele specific primer based CF assay for the Palestinian population, a technology that can be easily implemented in any diagnostic setting, including the 9 most frequent mutations, would result in a test that identifies 84% of the Palestinian mutations.

The differences in the rates of mutations identified in CF patients residing in the West Bank versus those in Gaza, are remarkable. For example, mutations c.1393-1G>A, p.(Gly85Glu), p.(Trp1282X), and p.(Asn1303Lys) were only present among Palestinians in the West Bank, while mutations p.(Lys684Serfs^*^38) and c.1585-1G>A were only present among Palestinians residing in Gaza (Figure 1). Interestingly, the p.(Phe508del) mutation was primarily prevalent among patients living in Gaza (68%) as compared to patients in the West Bank (32%). This may be due to the heterogeneous Arab population and the political barriers that were imposed on this population for many decades limiting their freedom of movement and concentrated the population into small communities in different geographic areas.

NGS technology combined with MLPA analysis proved to be a very efficient and cost-effective way to identify* CFTR* mutations. It should be noticed that heterozygous exon-spanning deletions or insertions are not observed with the PCR-based enrichment strategy followed by NGS analysis and therefore the combination of the NGS approach with MLPA analysis is highly recommended. The mutations included in the INNO-LIPA* CFTR*17 and* CFTR*19 kit cover only 66% of the mutations and therefore have a limited diagnostic value for the Palestinian population.

The relatively low mutation detection rate of 67% of the CF patients tested in this study is most likely caused by clinical misdiagnosis depending on the sweat chloride test. Values of this test can be influenced by nutritional state, skin condition, age, and many other factors, resulting in false positive sweat chloride values as high as 15% [20, 21]. All patients, in whom CF mutations have been identified and for whom a sweat chloride test has been performed, had a sweat chloride value higher than 70 mEq∖L. On the other hand, most patients without a mutation have sweat chloride values lower or around 60 mEq∖L and therefore have a less likely clinical diagnosis of cystic fibrosis. If we only consider the former group of patients, with a sweat chloride value higher than 70 mEq∖L, the mutation detection rate rises from 67 to 92%.

In conclusion, we identified the most common CF mutations and their respective frequency in the Palestinian population. Not only is this knowledge important for the families themselves but also it is a prerequisite to set up a reliable and sensitive diagnostic test for CF in this population. Genetic testing in this area for recessive disorders is highly recommended because of the high rates of consanguineous marriages among the Palestinians (25%–65%) [22], resulting in a high risk for genetic diseases including CF [23].

## Supplementary Material

Supplemental Table 1 shows the 28 sets of primers necessary to amplify the 27 exons of the *CFTR* gene. All primers are represented in the 5' to 3' direction. The maximum length of each PCR product is lower than 600 bp. For exon 14, two overlapping primersets were designed.

## Figures and Tables

**Table 1:** Patients data and sweat chloride concentrations.

#	Age (years)	Sex	Region	Sweat chloride values (mEq/L)
P1	1	M	Gaza	113
P2	3	M	Gaza	85
P3	8	M	Gaza	111
P4	11	M	Gaza	70
P5	11	F	Gaza	60
P6	5	F	Gaza	100
P7	8	F	Gaza	66
P8	3	M	Gaza	103
P9	<1	M	Gaza	81
P10	1	M	Gaza	NR
P11	7	M	Gaza	QNS
P12	10	M	Gaza	72
P13	8	M	Gaza	90
P14	4	M	Gaza	60
P15	6	M	Gaza	NR
P16	7.5	M	Gaza	90
P17	7	M	Gaza	96
P18	6	F	Gaza	76
P19	4	F	Gaza	114
P20	4	F	Gaza	118
P21	1	M	Gaza	77
P22	1	F	Gaza	114
P23	10	M	Gaza	100
P24	3	M	Gaza	126
P25	2	M	Gaza	129
P26	9	F	Gaza	105
P27	18	M	Gaza	42
P28	11	F	Gaza	128
P29	6	M	Gaza	135
P30	5	F	Gaza	QNS
P31	13	M	Gaza	106
P32	15	M	Gaza	60
P33	3	M	Gaza	107
P34	9	M	Gaza	46
P35	17	F	Gaza	64
P36	1.5	F	Gaza	129
P37	7	M	Gaza	NR
P38	2	F	Gaza	QNS
P39	13	F	Gaza	54
P40	10	M	Gaza	62
P41	4	M	Gaza	70
P42	7	M	West Bank	117
P43	18.5	M	West Bank	121
P44	5	M	West Bank	105
P45	13	M	West Bank	100
P46	12	M	West Bank	NR
P47	12	M	West Bank	110
P48	7	M	West Bank	108
P49	8	F	West Bank	105
P50	7	F	West Bank	NR
P51	15	F	West Bank	NR
P52	2	F	West Bank	NR
P53	10	F	West Bank	87
P54	12	F	West Bank	104
P55	2.5	F	West Bank	110
P56	12	F	West Bank	112
P57	31	M	West Bank	120
P58	3	F	West Bank	103
P59	15	F	West Bank	45
P60	30	M	West Bank	NR

NR: no result, used to indicate nonreported results.

QNS: quantity not sufficient, used to indicate insufficient sweat samples to perform the sweat test.

**Table 2:** *CFTR* mutations among Palestinians in Gaza and West Bank.

c-notation	p-notation	Exon/intron	dbSNP #	# of patients Gaza	# of patients West Bank	Patient(s)
del exon2		Exon 2		1	1	24 (het), 43
c.223C>T	p.(Arg75^*^)	Exon 3	121908749	1		13
c.254G>A	p.(Gly85Glu)	Exon 3	75961395		4	44 (het), 56, 57 (het), 58
c.1040G>C	p.(Arg347Pro)	Exon 8	77932196	1		33
c.1209+1G>A		Intron 9	397508176	1		2
c.1393−1G>A		Intron 10	397508200		4	42, 47, 54, 55 (het)
c.1521_1523delCTT	p.(Phe508del)	Exon 11	113993960	11	5	1, 3, 9, 11, 17, 19, 22 (het), 23, 24 (het), 28 (het), 29, 46, 48, 49 (het), 50, 52
c.1585−1G>A		Intron 11	76713772	2		26, 31
c.1624G>T	p.(Gly542^*^)	Exon 12	113993959	1		28 (het)
c.2051_2052delAAinsG	p.(Lys684Serfs^*^38)	Exon 14	121908799	2		8, 25
c.2089_2090insA	p.(Arg697fs)	Exon 14	397508341	1		22 (het)
c.2988+1Kbdel8.6Kb		Exons 19, 20, 21		4	1	10, 20, 21, 36, 49 (het)
c.3299A>C	p.(Gln1100Pro)	Exon 20	397508535		1	57 (het)
c.3793G>A^1^	p.(Gly1265Arg)	Exon 23			1	16
c.3808G>A	p.(Asp1270Asn)	Exon 23	11971167		1	18 (het)
c.3846G>A	p.(Trp1282^*^)	Exon 23	77010898		2	55 (het), 60
c.3909C>G	p.(Asn1303Lys)	Exon 24	80034486		2	44 (het), 51
c.4251delA	p.(Glu1417fs)	Exon 27	397508706	1		6

^1^Previously undescribed mutation.

All mutations are homozygous except those indicated by (het).

In patients P4, P5, P7, P12, P14, P15, P27, P30, P32, P34, P35, P37, P38, P39, P40, P41, P45, P53, and P59 no mutation was detected.

In P18 only one mutation was detected.
