# Supplementary material for: Next Generation Sequencing to Determine the Cystic Fibrosis Mutation Spectrum in Palestinian Population
Source: Dis Markers. 2015 Jan 26;2015:458653. doi: 10.1155/2015/458653 (PMC4321085; doi:10.1155/2015/458653)
Supplement: Supplementary file 1 — Supplemental Table 1 shows the 28 sets of primers necessary to amplify the 27 exons of the CFTR gene. All primers are represented in the 5' to 3' direction. The maximum length of each PCR product is lower than 600 bp. For exon 14, two overlapping primersets were designed. [file 458653.f1.docx]

**Supplemental table 1:** Primers used to amplify *CFTR* gene exons. All primers are presented from 5’ to 3’.

| **Exon #** | **Forward Primer** in | **Reverse Primer** | **Amplicon Length (bp)^*^** |
| --- | --- | --- | --- |
| 1 | GAAAGCCGCTAGAGCAAA | CCAAACCCAACCCATACA | 419 |
| 2 | GCCTGTAAGAGATGAAGCCT | CCACCATACTTGGCTCCTAT | 435 |
| 3 | TCTGGCTGAGTGTTTGGT | TGGTTTCTTAGTGTTTGGAGTTG | 448 |
| 4 | TTGTGTTGAAATTCTCAGGGTAT | CTTGTACCAGCTCACTACCTA | 414 |
| 5 | CAACTAGAAGCATGCCAGTAT | TAATTACTATTATCTGACCCAGGAA | 428 |
| 6 | AGAACCACGAAGTGTTTGA | CACTGAAGATCACTGTTCTATGC | 427 |
| 7 | AGGCTGTCATAAGGGATAGAG | AGGTGGAAGTCTACCATGA | 481 |
| 8 | TCCATTCCAAGATCCCTGATA | CCATCATACTGTCCAGAGAAA | 550 |
| 9 | AGATGTAGCACAATGAGAGTAT | TGGCCATTCCTCTACTTCTTA | 388 |
| 10 | TCCTCTAGAAACCGTATGCT | CTTCCAGCACTACAAACTAGA | 483 |
| 11 | CTTCTGCTTAGGATGATAATTGG | GCTTACCCATAGAGGAAACA | 436 |
| 12 | TCAACTGTGGTTAAAGCAATAG | GATTCTTAACCCACTAGCCATA | 391 |
| 13 | TCTACACTAGATGACCAGGAA | GAGAAACTGGTTTAGCATGAG | 410 |
| 14a | CTATCAGAATTCACAAGGTACCAAT | AGAGTTGATTGGATTGAGAATAGAA | 573 |
| 14b | GTCTCCTGGACAGAAACAA | TTTAAGATACACCTTATCCTAATCCT | 616 |
| 15 | CCACAATGGTGGCATGAAA | AGTAGTGGTTCTACTTGTTGATT | 480 |
| 16 | GGGAGGAATAGGTGAAGATG | CTGCACATGCTCACAATTTA | 486 |
| 17 | AAGGGTGCATGCTCTTCTA | TGATGGTGGATCAGCAGTT | 459 |
| 18 | TTCTAAGTCTATCTGATTCTATTTG | GGGATTGCCTCAGGTTTG | 477 |
| 19 | CACTGACACACTTTGTCCAC | CCATGTGTACTTTGTAATATAGTTTCCT | 436 |
| 20 | AGAATGGCACCAGTGTGAA | GGAAATTCAAAGAAATCACTTGTTC | 572 |
| 21 | GCCCTAGGAGAAGTGTGAATA | GAATGCTCACTGCAGTATTAGAT | 438 |
| 22 | GTGAAATTGTCTGCCATTCTTAAA | GGTTCAGGACTCTGCAAATTAAA | 527 |
| 23 | TCCACTGGTGACAGGATAAA | AAAGACAGCAATGCATAACAAAT | 432 |
| 24 | CAAGGGACTCCAAATATTGCT | AGCCATTTGTGTTGGTATGAG | 405 |
| 25 | TTCAAATGGTGGCAGGTAGT | TCTGTTCCCACTGTGCTATT | 395 |
| 26 | AAGAAGTACTGGTGATTCTACAT | AGAATTACAAGGGCAATGAGAT | 456 |
| 27 | GTCTGACCTGCCTTCTGTC | AGACCCATATCAGTGTCCTC | 466 |

^*^bp: base pairs
